# Current landscape and research gaps in artificial intelligence for ophthalmic nursing: a scoping review

**DOI:** 10.3389/fmed.2026.1943007

**Published:** 2026-09-03

**Authors:** Qiong Song, Xiaofang Huang, Yamin Hu, Kaiqin She

**Affiliations:** Department of Ophthalmology, West China Hospital, Sichuan University, Chengdu, China

**Keywords:** artificial intelligence, augmented reality, machine learning, nursing education, ophthalmic nursing, ophthalmology, scoping review, triage

## Abstract

**Introduction:**

Artificial intelligence (AI) is advancing rapidly across clinical medicine, yet its application within ophthalmic nursing, spanning emergency triage, perioperative care, day surgery, and patient education, remains poorly characterized. The growing burden of eye disease and the demand for community-based ophthalmic screening create an urgent context for understanding where AI can augment nursing capacity in this field and what barriers may be limiting evidence generation. This review aimed to systematically map published evidence on AI applications in ophthalmic nursing practice and education, characterize the technologies deployed and outcomes reported, and identify recurring research gaps and barriers through interpretive synthesis of the mapped literature.

**Methods:**

Four electronic databases (Web of Science Core Collections, PubMed, Scopus, and CINAHL) were searched from inception to June 12, 2026. Peer-reviewed English-language articles simultaneously addressing AI or machine learning, ophthalmology or eye conditions, and nurse engagement were eligible. Records were independently screened by two reviewers and data were extracted using a standardized charting form.

**Results:**

From 254 records retrieved, 7 studies met the inclusion criteria (2023–2026). Applications spanned emergency triage and primary diagnosis, surgical nursing assistance (including one pre-clinical prototype without direct nurse involvement), day ward workflow management, and nursing education. Reported evaluation domains included triage or diagnostic performance, engineering detection performance, usability and acceptability, workflow efficiency, and educational outcomes. AI paradigms included classical machine learning, deep learning (convolutional neural networks), and large language models, deployed via clinical workflow interfaces, augmented reality, and robotic systems, and processing structured and multimodal data (image, text, audio).

**Discussion:**

Despite ophthalmology's prominent role in the early development of clinical AI, dedicated research on AI in ophthalmic nursing remains uncommon in the published literature. To our knowledge, this is the first scoping review to draw on international, English-language sources on this topic. Future research should prioritize nurse-led studies in community and primary care settings, broader modality exploration matched to nursing tasks, rigorous prospective evaluation of nursing-specific outcomes, and, where no current study exists, nurses as active co-designers of AI tools.

## 1 Introduction

Vision impairment and blindness affect at least 2.2 billion people worldwide, generating substantial and growing demand for ophthalmic screening, triage, treatment, and follow-up services (1). Within ophthalmic healthcare systems, nurses serve a critical function across the full care continuum, from community-based and primary care screening and patient triage through to perioperative nursing, day surgery management, postoperative follow-up, and patient education. The exact distribution of these frontline roles varies across health systems: in some settings, functions such as first-contact assessment, triage, and screening are undertaken by ophthalmic nurses, whereas in others they are performed more often by optometrists, ophthalmic technicians, or other allied eye-care personnel. However, the nursing shortage has remained a persistent global issue. The World Health Organization recommends a minimum ratio of three nurses per 1,000 population for the general nursing workforce, a benchmark unmet in many health systems (3). In high-volume ophthalmic emergency settings, a substantial proportion of attendances involve presentations that do not require urgent specialist intervention, placing unnecessary burden on specialist resources and triage nurses (4). Meanwhile, in surgical and day ward environments, the pace and volume of procedures demand workflows that are both efficient and reliably error-resistant. Notably, the growing demand for ophthalmic screening at the primary care and community level places particular pressure on nursing workforces that often lack specialist ophthalmic training, creating a structural gap between population need and clinical capacity (5).

Artificial intelligence (AI) and machine learning have demonstrated transformative potential across clinical medicine, and ophthalmology was among the first specialties to experience this transformation. In 2016, Gulshan and colleagues demonstrated that a deep convolutional neural network could detect diabetic retinopathy from fundus photographs with specialist-level accuracy, marking a landmark advance that signaled a new era for AI in ophthalmic screening (6). Since then, AI applications in ophthalmic imaging have expanded rapidly to encompass diabetic retinopathy screening, glaucoma detection, and age-related macular degeneration monitoring (7–9). Yet despite this momentum, the intersection of AI and nursing practice within ophthalmology where nurses are the primary actors in triage, care delivery, and patient education has received comparatively little systematic attention.

Scoping reviews offer a structured methodology for mapping available evidence across heterogeneous and emerging research areas, identifying knowledge gaps, and informing future research priorities (10). To our knowledge, no scoping review has examined AI applications specifically within the domain of ophthalmic nursing based on international, English-language sources. Accordingly, this review asked the following question: what published evidence exists on AI applications involving nurse engagement in ophthalmic clinical practice and education, and what research gaps and recurring barriers emerge across settings, nurse roles, technologies, and reported outcome domains? Thus, we conducted this review to systematically identify and characterize the published evidence on AI applications in ophthalmic nursing, interpret recurring barriers and research gaps emerging from the mapped literature, and derive recommendations for future research priorities and funding directions.

## 2 Methods

This scoping review was conducted following the methodological framework proposed by Arksey and O'Malley (10) and reported in accordance with the Preferred Reporting Items for Systematic Reviews and Meta-analyses Extension for Scoping Reviews (PRISMA-ScR) (12).

### 2.1 Search strategy and selection criteria

Four databases were searched from inception to June 12, 2026: Web of Science Core Collections, PubMed, Scopus, and CINAHL Complete. Database specific search strategies were developed for each platform, combining controlled vocabulary terms (including MeSH headings where applicable) and keywords related to AI or machine learning, ophthalmology or eye and vision conditions, and nursing. Ophthalmology terms were applied broadly across title, abstract, and keyword fields; AI and nursing terms were applied in title and keyword fields in Web of Science, PubMed, and Scopus, and additionally in abstract and subject heading fields in CINAHL. Boolean operators (AND, OR) were used throughout. Full search strategies for all databases are provided in the Supplementary Material.

Studies were included if they met all three of the following criteria: (1) addressed AI or machine learning; (2) addressed ophthalmology or conditions affecting eye health and vision; and (3) involved nurse engagement. We defined nurse engagement broadly to include four distinct forms: (a) nurses as direct end-users of AI systems; (b) nursing students as intervention participants; (c) nurses as performance benchmarks or comparators for AI evaluation; or (d) nursing roles that the AI system was designed to support or replicate. For studies in which nurses served solely as statistical performance benchmarks with no direct nurse-AI interaction during the study, and studies designing AI systems to replicate a specific nursing role, they were retained as eligible on the grounds that these performance measurements constitutes a direct assessment of AI's potential implications for nursing practice. Additional criteria required English language and peer-reviewed publication as a journal article or review. Studies were excluded if full text was unavailable; if only one or two of the three required themes were addressed; if the research involved animal subjects; if the language was not English; or if the document was a conference paper, editorial, letter, or other non-peer-reviewed item.

All records were imported into EndNote for deduplication. Two reviewers independently screened records by title and abstract, followed by full-text assessment of eligible records. Disagreements were first addressed through discussion between the two reviewers; any unresolved disagreements were adjudicated by a third senior reviewer. Forward and backward citation tracking of all included studies were also done for additional eligible studies.

### 2.2 Data charting

A standardized data charting form was jointly developed and used to extract the following from each included study: first author, year of publication, journal, country of origin, study design, clinical or educational setting, target population or condition, data modality (text, image, audio, or multimodal), AI paradigm and specific algorithms, AI task type, and key outcomes with performance metrics. Data charting was performed independently by two reviewers; disagreements were first addressed through discussion, and any remaining disagreements were adjudicated by a third senior reviewer. Given the heterogeneity of study designs and outcomes, a narrative synthesis approach was adopted, with studies grouped by clinical domain and AI technology type. During screening and charting, the reviewers also noted recurring exclusion patterns and contextual features relevant to why AI, ophthalmology, and nursing literatures rarely intersect; these observations informed the interpretive discussion of structural barriers, but were not tabulated as a formal quantitative charting domain.

## 3 Results

### 3.1 Study selection

**Figure 1:**
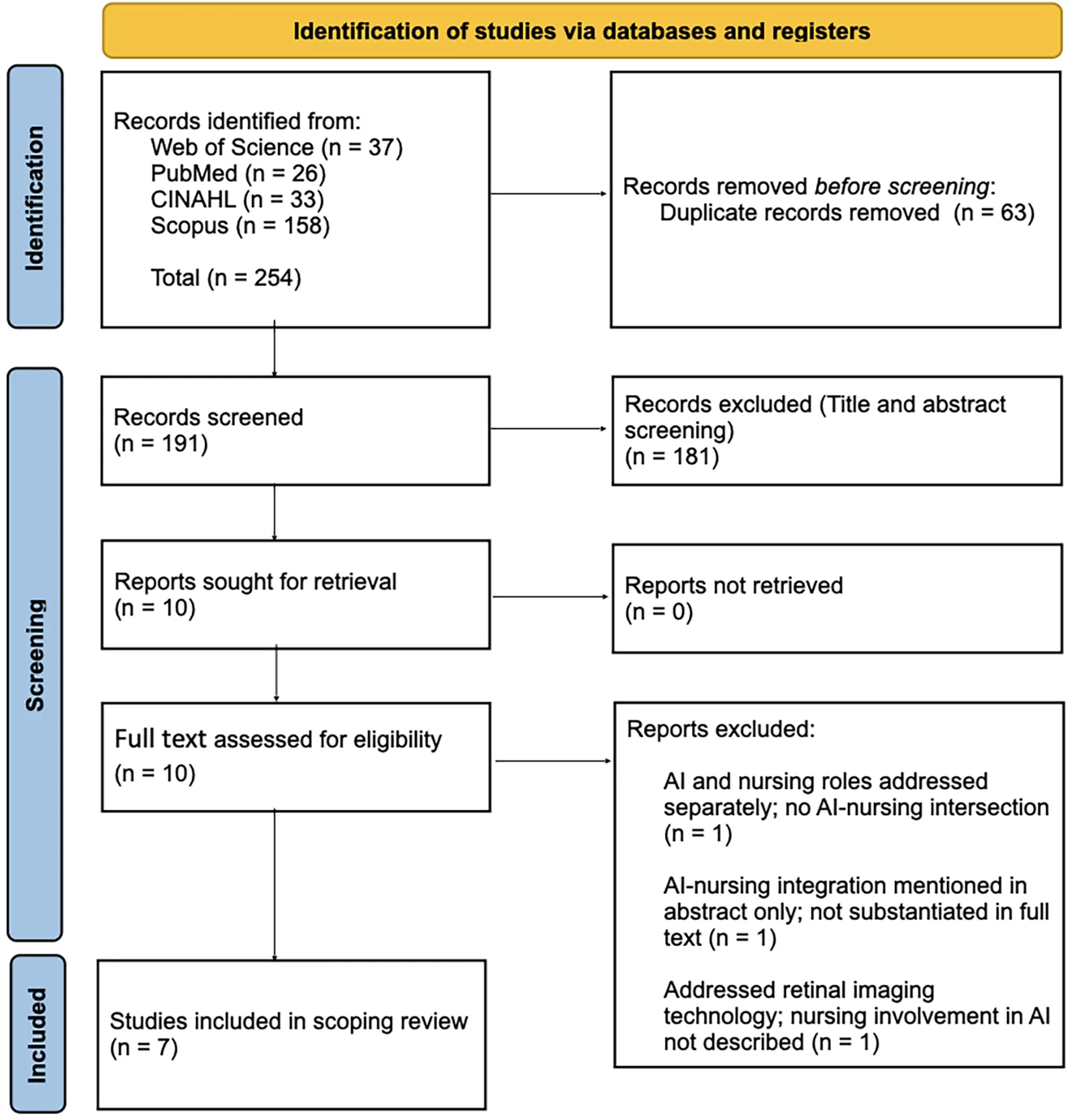
PRISMA-ScR flow diagram of study selection.

The search retrieved 254 records: Web of Science Core Collections (*n* = 37), PubMed (*n* = 26), Scopus (*n* = 158), and CINAHL (*n* = 33). After deduplication, 191 records underwent title and abstract screening, with 181 excluded at that stage. Ten records were assessed in full text; three were excluded because they did not address all three required thematic domains. Seven studies met all eligibility criteria and were included (Figure 1).

### 3.2 Characteristics of included studies

**Figure 2:**
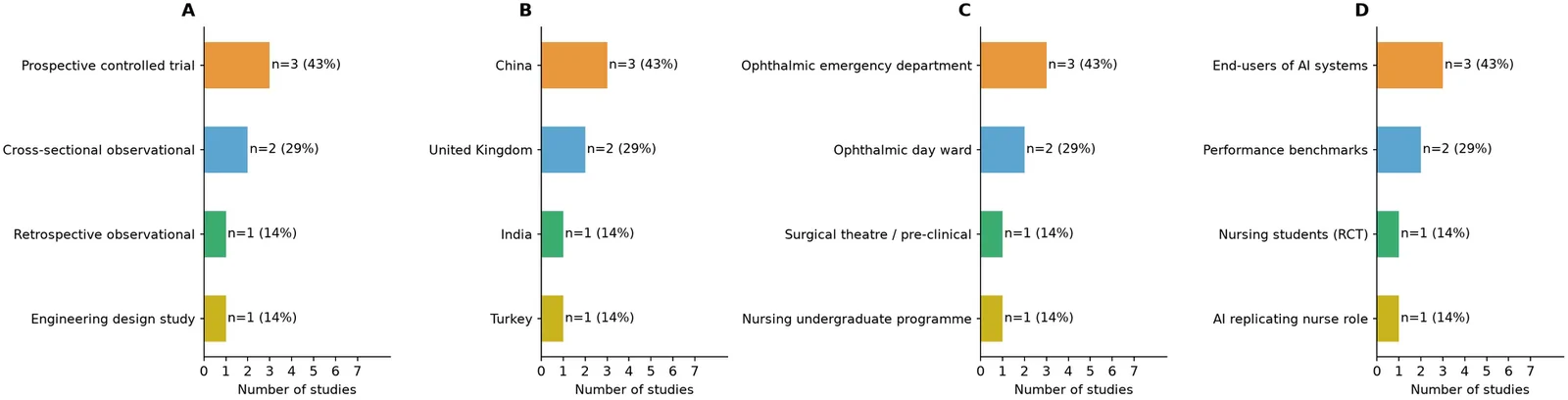
Distribution of seven included studies across four key characteristics. Panel **(A)**: study design; Panel **(B)**: country of origin; Panel **(C)**: department or clinical setting; Panel **(D)**: form of nurse engagement with the AI system. Bars represent number of studies; percentages are shown in parentheses. All seven included studies were conducted in tertiary academic hospitals or academic university settings; institution type is therefore not displayed as a separate panel. AI, artificial intelligence; RCT, randomized controlled trial.

The seven included studies were published between 2023 and 2026 (Tables 1, 2). Countries of origin included China (*n* = 3) (16, 18, 20), the United Kingdom (*n* = 2) (15, 17), Turkey (*n* = 1) (14), and India (*n* = 1) (13). All seven studies were conducted in tertiary academic hospitals or academic universities. Study designs were heterogeneous: three prospective controlled trials (14, 18, 20) (including one randomized controlled trial (14)), two cross-sectional observational studies (16, 17), one retrospective observational study (15), and one engineering design and development study (13). Settings spanned ophthalmic emergency departments (*n* = 3) (15–17), ophthalmic day wards (*n* = 2) (18, 20), surgical theatre (*n* = 1, development stage only) (13), and undergraduate nursing education (*n* = 1) (14) (Figure 2).

**Table 1:** Characteristics of included studies.

Study	Year	Country and region	Design	Setting	Clinical domain	AI application	AI technology	Data modality	Nurse engagement	Key findings
Brandao-de-Resende et al. (15)	2023	UK/Brazil	Retrospective observational	Ophthalmic ED	Triage	AI-assisted emergency triage (DOTS)	Classical ML: logistic regression, decision tree, random forest, XGBoost	Text	Triage nurses as performance benchmark^a^	Nurse sensitivity 96.4% (95% CI 94.5–97.7) vs. AI 94.5% (95% CI 92.3–96.1) (nurses higher); AI specificity 42.4% (95% CI 38.8–46.1) vs. nurse 25.1% (95% CI 22.0–28.5) (AI 17.3 percentage points higher); robust on external validation
Chen et al. (16)	2023	China	Cross-sectional observational	Ophthalmic ED	Triage & primary diagnosis	Multimodal AI for triage and diagnosis (EE-Explorer)	CNN (YOLOv3, DenseNet201) + XGBoost	Multimodal (text + image)	Triage performance compared with clinical nurses^a^	Three-level triage (macro-averaged AUC 0.982 [95% CI 0.966–0.998]); outperformed nurses (*p* < 0.001); diagnostic classification accuracy 0.808 (95% CI 0.776–0.840); robust external validation (4 hospitals)
Jindal et al. (17)	2023	UK	Cross-sectional mixed methods	Ophthalmic ED	Triage	Usability of AI triage platform (DOTS)	Classical ML (as above)	Text	Ophthalmic nurses among end-user participants	90% easy to use; 85% safe; 95% fast; 95% willing to use in clinical workflow
Huang et al. (18)	2024	China	Prospective controlled trial	Ophthalmic day ward	Day ward nursing practice	AR-AI nursing assistant (AR-IAS)	Augmented reality (CV, NLP)	Multimodal (image + audio + text)	Ophthalmic day ward nurses as end-users	SUS score 75.83/100 (good; (19) benchmark); no significant difference vs. PDA system
Rekha and Kaliyappan (13)	2024	India	Engineering design study	Development stage only	Surgical nursing assistance	Robotic scrub nurse for cataract surgery (CRASCS)	Robotics + CV (YOLO) + NLP	Multimodal (image + audio)	System designed to replicate scrub nurse role	Instrument and location detection Precision of 0.915, Recall of 0.864, mAP50 0.904; surgery phase identification demonstrated
Lin et al. (20)	2025	China	Prospective controlled trial	Outpatient Ophthalmology clinic/Ophthalmic day ward	Surgical nursing assistance	AR-AI surgical information verification	Augmented reality (YOLOv5 + NLP)	Multimodal (image + audio + text)	Surgical nurses as end-users	Accuracy not significantly different from manual verification (95.33% vs. 98.67%; *p* = 0.087); 11 s faster per patient (*P* < 0.001); ∼4,680 pages paper saved/year
Dogan and Yilmaz (14)	2026	Turkey	Prospective randomized controlled trial	Nursing undergraduate program	Nursing education	AI chatbot for dry eye syndrome education	Large language model (Gemini 2.0 Flash)	Text	Undergraduate nursing students	Similar knowledge acquisition vs. web learning; chatbot superior in content quality, readability, understandability, and actionability

AI, artificial intelligence; AR, augmented reality; AR-IAS, AR-based intelligent assistance system; AUC, area under the receiver operating characteristic curve; CNN, convolutional neural network; CRASCS, collaborative robot acting as a scrub nurse for cataract surgery; CV, computer vision; DOTS, DemDx Ophthalmology Triage System; ED, emergency department; 5-DOF, five degrees of freedom; mAP50, mean average precision at 0.50 intersection-over-union threshold; ML, machine learning; NLP, natural language processing; PDA, personal digital assistant; SUS, system usability scale.

a The experience level and specialist qualification status of comparator triage nurses were not reported in either study.

**Table 2:** Evaluation domains, comparators, and validation approaches across included studies.

Study	Primary evaluation domain	Reference standard or comparator	Validation/evaluation basis	Key reported outcome
Brandao-de-Resende et al. (15)	Triage performance	Triage nurses; confirmed diagnosis-based urgency classification	Temporal validation and external validation (Brazil center)	Temporal: Sensitivity 94.5% (95% CI 92.3–96.1) vs. nurses 96.4% (95% CI 94.5–97.7); specificity 42.4% (95% CI 38.8–46.1) vs. nurses 25.1% (95% CI 22.0–28.5) External: Sensitivity 95.2% (95% CI 92.5–97.0); specificity 32.2% (95% CI 27.4–37.0)
Chen et al. (16)	Triage and primary diagnostic performance	Triage nurses for triage comparison; ophthalmologist-labeled data for model development/testing	Internal testing plus external testing at 4 hospitals	Triage macro-AUC 0.982 (95% CI 0.966–0.998); external triage AUC 0.988 (95% CI 0.967–1.000); primary diagnosis CA 0.808 (95% CI 0.776–0.840) internal, 0.718 (95% CI 0.644–0.792) external
Jindal et al. (17)	Usability/acceptability of DOTS platform	Clinician end-users, including ophthalmic nurses	Think-aloud interview and usability questionnaires	85% safe; 95% fast; 95% willing to use in workflow
Huang et al. (18)	Usability/acceptability in day ward nursing	Existing PDA-based system	Controlled usability comparison with nurse users	SUS 75.83/100; no statistically significant difference vs. PDA
Rekha and Kaliyappan (13)	Engineering detection performance	Instrument and phase-recognition technical benchmarks	Pre-clinical development study	Precision 0.915; Recall 0.864; mAP50 0.904
Lin et al. (20)	Workflow efficiency and verification accuracy	Manual verification process	Prospective controlled clinical comparison (*n* = 300)	No significant accuracy difference; 11-second time saving per patient
Dogan and Yilmaz (14)	Educational outcomes	Web-based instruction	Randomized controlled trial (*n* = 63)	Similar knowledge gain; better content quality, readability, understandability, and actionability

CA, classification accuracy; AUC, area under the receiver operating characteristic curve; PDA, personal digital assistant; SUS, system usability scale.

Nurse engagement took five distinct forms: nurses as direct end-users of AI systems (*n* = 3) (17, 18, 20), nurses as performance benchmarks for AI evaluation (*n* = 2) (15, 16), nursing students as intervention participants (*n* = 1) (14), nurses as the clinical role that the AI system was designed to replicate (*n* = 1) (13), and nurses as active co-designers or co-investigators in AI development (*n* = 0) (Figure 2).

### 3.3 Clinical domains addressed

The seven included studies addressed two broad nursing domains: clinical nursing practice (*n* = 6) and ophthalmic nursing education (*n* = 1). Within clinical nursing practice, three sub-domains emerged: triage and primary diagnosis, surgical nursing assistance, and day ward nursing practice.

#### 3.3.1 Triage and primary diagnosis (*n* = 3)

Three studies applied AI to the ophthalmic emergency triage process. Two of these reports concerned the same underlying DemDx Ophthalmology Triage System (DOTS): Brandao-de-Resende and colleagues (15) reported model development and validation, whereas Jindal and colleagues (17) reported clinician usability and acceptability of the same platform. DOTS, a classical ML classifier that processes structured form data to generate one of three triage recommendations, achieved a sensitivity of 94.5% (95% CI 92.3–96.1) and a specificity of 42.4% (95% CI 38.8–46.1) on temporal validation; the comparator triage nurses achieved a sensitivity of 96.4% (95% CI 94.5–97.7) and a specificity of 25.1% (95% CI 22.0–28.5) on the same dataset (15). In emergency triage, sensitivity (the ability to identify all patients requiring urgent care) is the clinically paramount metric; the DOTS results therefore represent a specificity trade-off rather than an overall performance advantage over nurses. DOTS performed consistently on external validation at an independent center (15). Clinician evaluation (*n* = 20) found 85%–95% of participants, including ophthalmic nurses, rated DOTS safe, fast, and suitable for clinical workflow (17). The third study developed EE-Explorer, a multimodal system combining ocular images (smartphone and slitlamp) and clinical metadata, achieving a macro-averaged AUC of 0.982 (95% CI 0.966–0.998) for three-level emergency severity classification (urgent, semiurgent, and non-urgent) that significantly exceeded nurse performance (*p* < 0.001), with external validation across four independent hospitals (16). The experience level and specialist qualification status of the comparator triage nurses were not reported in either study; Brandao-de-Resende and colleagues (15) explicitly acknowledged this as a limitation of their study design.

#### 3.3.2 Surgical nursing assistance (*n* = 2)

Lin and colleagues (20) developed AR smart glasses for pre-operative surgical information verification: accuracy was not significantly different from manual verification methods (95.33% vs. 98.67%; *p* = 0.087), with 11 s saved per patient encounter. Statistical non-significance at this sample size (300 participants) does not establish clinical equivalence; formal non-inferiority testing with a pre-specified acceptable margin would be required before deployment. Rekha and Kaliyappan (13) designed CRASCS, a pre-clinical robotic scrub nurse system for instrument detection and supply in cataract surgery, achieving Precision of 0.915, Recall of 0.864, and mAP50 (mean average precision at 0.50 intersection-over-union threshold) of 0.904, motivated by global surgical nursing shortages.

#### 3.3.3 Day ward nursing practice (*n* = 1)

Huang and colleagues (18) developed AR-IAS, an AR assistant for patient identification, medication order confirmation, patient education delivery, and surgical transport management in ophthalmic day wards. The system achieved good usability (SUS (System Usability Scale) = 75.83/100 (19); benchmark scale) with no statistically significant difference from an existing PDA-based system.

#### 3.3.4 Nursing education (*n* = 1)

Dogan and Yilmaz (14) randomized 63 undergraduate nursing students to AI chatbot versus web-based instruction for dry eye syndrome education. Both groups achieved similar post-intervention knowledge gains; the AI chatbot was rated significantly superior in educational content quality, readability, and actionability.

### 3.4 AI approaches, data modalities, and deployment platforms

The seven studies varied across three main dimensions: the underlying AI paradigms, the processed data modalities, and the platforms through which the systems were deployed (Table 1). The studies can be grouped into four distinct AI approaches:

**Classical machine learning (*n* = 2):** Both DOTS studies (15, 17) applied ensembles of classical algorithms (logistic regression, decision tree, random forest, XGBoost). These models processed structured clinical text data (modality) and were deployed via standard decision-support software applications (platform) for ophthalmic emergency triage.

**Computer vision and natural language processing (*n* = 3):** Three studies utilized a combination of computer vision (CV) and natural language processing (NLP) to process multimodal data (image, audio, and text), though they differed in their deployment platforms. Two studies (18, 20) from the same institution deployed facial recognition, surgical marking interpretation, and voice-activated queries via augmented reality (AR) smart glasses to support nursing tasks in surgical and day ward settings. Another study (CRASCS) (13) utilized YOLO-based instrument detection and speech recognition deployed through a 5-DOF (5-degree-of-freedom) robotic arm to replicate surgical scrub nurse tasks.

**Hybrid deep learning and classical machine learning (*n* = 1):** EE-Explorer (16) combined deep learning architectures (YOLOv3, DenseNet201) for image-based feature extraction with XGBoost for text metadata. This system processed multimodal data (slitlamp and smartphone imaging combined with structured clinical text) and was delivered as a software system.

**Large language models (*n* = 1):** One study (14) employed a generative AI system using a commercial LLM (Gemini 2.0 Flash). This model processed purely text data and was deployed through an interactive software interface for personalized text-based nursing education.

### 3.5 Sample sizes and external validation

**Sample sizes:** Study populations varied widely. The two DOTS studies (15, 17) included 11,315 (training) and 20 (usability evaluation) participants respectively. EE-Explorer (16) trained on 2,038 (triage) and 2,405 (diagnosis) patients. The AR studies involved 300 patients (20) and a small nursing cohort (15 nurses) (18) respectively. The RCT enrolled 63 students (14). The engineering study (13) did not report a clinical sample size.

**External validation:** Only two studies included external validation of AI model performance. Brandao-de-Resende and colleagues (15) validated DOTS at an independent tertiary center in Brazil (*n* = 761). Chen and colleagues (16) validated EE-Explorer at four external hospitals (*n* = 103). The remaining five studies did not include external validation, instead relying on internal testing, single-site controlled designs, or subjective usability evaluation.

### 3.6 Evaluation domains across studies

The included studies assessed AI using five distinct evaluation domains (Table 2), underscoring why direct cross-study comparison is limited. Two studies emphasized triage or diagnostic performance against clinical comparators or expert-labeled reference standards (15, 16). One pre-clinical engineering study emphasized technical detection performance through precision, recall, and mAP50 for instrument recognition rather than patient-level clinical outcomes (13). Two studies primarily reported usability and acceptability outcomes through questionnaire-based evaluation of clinician experience (17, 18). One study focused on workflow efficiency and verification accuracy in a real clinical process (20). The educational trial assessed knowledge acquisition and content quality-related educational outcomes rather than clinical performance (14). For this reason, the evidence base is best interpreted as a heterogeneous map of early applications rather than a set of directly comparable effectiveness studies.

## 4 Discussion

The seven studies included in this scoping review collectively demonstrate that AI has begun to enter ophthalmic nursing contexts across a range of clinical and educational functions. Yet the most striking finding of this review is how few of them exist. Despite the sustained global expansion of AI research in clinical medicine since the early 2010s, and despite ophthalmology's prominent place in that history, as evidenced when Gulshan and colleagues published the first landmark application of deep learning to clinical diagnosis for diabetic retinopathy from fundus photographs in 2016 (6), only seven peer-reviewed English-language studies met our criteria for AI in ophthalmic nursing. This scarcity stands in sharp contrast to the explosive growth of AI research in medicine more broadly, and to the substantial and growing demand for ophthalmic services that nurses are positioned to address. Understanding this gap, and what it will take to close it, is the central challenge this review seeks to articulate.

### 4.1 Current evidence: scope and significance

The included studies capture a meaningful breadth of potential applications. Three studies examined AI triage support in ophthalmic emergency departments, the most mature area, where ML-based systems demonstrated triage performance that was complementary to, rather than uniformly superior to, that of triage nurses (15–17). The DOTS system achieved 17.3 percentage points higher specificity than triage nurses (42.4% [95% CI 38.8–46.1] vs. 25.1% [95% CI 22.0–28.5]), but at the cost of slightly lower sensitivity (94.5% [95% CI 92.3–96.1] vs. 96.4% [95% CI 94.5–97.7])—a performance profile that may reduce unnecessary specialist referrals but which requires careful clinical validation before deployment in time-critical triage settings where sensitivity is paramount (15). EE-Explorer achieved a macro-averaged AUC of 0.982 (95% CI 0.966–0.998, three-level severity classification) that significantly exceeded nurse performance (16), although the comparator nurses’ experience and subspecialty qualifications were not reported. High clinician acceptance rates in the DOTS usability study (17) suggest initial acceptability of the platform, but do not in themselves establish implementation readiness. Two studies from a single Chinese institution advanced AI-assisted nursing workflow in day surgery and day ward settings using AR technology (18, 20) and one study demonstrated the feasibility of an AI chatbot for targeted ophthalmic nursing education (14). Taken together, these findings suggest preliminary integration of AI across several points in the ophthalmic nursing care continuum, from pre-operative safety checks to emergency decision support and professional education.

Despite the breadth across seven studies, the independence of the evidence base is limited. Two of three triage studies (15, 17) evaluated the same underlying DOTS system, and two workflow studies (18, 20) originated from a single institution. The effective contribution to unique research questions is therefore closer to four independent groups than to seven studies, reinforcing the overall conclusion that the evidence base is at an early, exploratory stage.

The practical implications vary by domain. For triage applications, the evidence is mature to justify prospective clinical trials evaluating safety, workflow integration, and patient outcomes. For AR-based nursing workflow tools, the proof-of-concept results are encouraging, but no study examined patient safety outcomes, medication error rates, or staff cognitive load, which are all essential outcomes for evaluating real-world nursing impact. For nursing education, the AI chatbot's advantage in educational quality and actionability over web-based learning aligns with broader trends in personalized digital health education and suggests potential for further development and evaluation in clinical nursing education settings.

### 4.2 Why so few studies? Research gaps and structural barriers

The scarcity of included studies may be attributed to three recurring patterns suggested by the mapped evidence and the exclusion patterns observed during screening. These barriers were not formally pre-specified or systematically extracted as quantitative variables, but emerged consistently across the literature, the recurring reasons why adjacent studies fell outside scope, and the settings in which eligible studies were concentrated.

**First recurring observation: most AI-in-ophthalmology research excludes nursing.** The dominant paradigm in ophthalmic AI research is the comparison of algorithmic performance against ophthalmologist, typically using fundus images, optical coherence tomography, or other imaging modalities to screen for retinal diseases. In these studies, nursing is neither the target beneficiary nor a measured variable. A study evaluating AI-based detection of diabetic retinopathy against general practitioner performance, for example, would not address the nursing workforce at all, even though nurses frequently coordinate screening programs (21). Similarly, studies examining ethical and governance dimensions of AI in ophthalmic screening, such as those addressing transparency and data privacy in diabetic retinopathy programs, are outside the scope of ophthalmic nursing if nurses are not the actors studied (22). The ophthalmic AI literature is thus rich, but overwhelmingly physician-centric.

**Second recurring observation: most AI-in-nursing research excludes ophthalmology.** Conversely, the AI and nursing literature has grown substantially in recent years, but tends to address nursing competencies, attitudes, or workflows in general or non-ophthalmic contexts. For instance, research evaluating LLMs on national nursing licensing examinations in Japan, where ophthalmology constitutes only a subset of examination content, captures AI in nursing education broadly, but not ophthalmic nursing specifically (23). Similarly, studies examining AI in chronic disease nursing that include diabetic retinopathy as one outcome measure among many are excluded from our review if ophthalmic nursing is not the explicit focus (24). The result is that the two large literatures, AI in ophthalmology and AI in nursing, rarely converge.

**Third possible explanation: the definition of “nurse engagement” is structurally variable across health systems.** In many countries and regions, there are no specialist ophthalmic nurses, and ophthalmic triage is not a nurse-led function (30). Where nursing roles in ophthalmic care are limited, AI tools designed to augment those roles will not be developed or evaluated. The geographic concentration of included studies, predominantly from the United Kingdom and China, partly reflects the presence of well established ophthalmic nursing workforces in these settings, particularly the nurse-led emergency triage model at institutions such as Moorfields Eye Hospital.

### 4.3 AI technology lag and research opportunities

The AI technologies employed in the included studies illustrate a narrower range of modalities and nurse-centered use cases than is seen in ophthalmic AI more broadly. The deep learning revolution began in earnest in 2012 with breakthrough performance on the ImageNet challenge (26), and Gulshan and colleagues demonstrated clinical-grade retinal AI in 2016 (6). Landmark validations followed rapidly: Ting and colleagues demonstrated specialist-level diabetic retinopathy detection in a multiethnic population in 2017 (8) and De Fauw and colleagues demonstrated clinically applicable deep learning for 3D OCT based retinal diagnosis in 2018 (9). This pattern should not be interpreted simply as technological backwardness: for structured triage data, classical machine learning may remain an appropriate and practical choice. Rather, the striking feature is that published ophthalmic nursing studies have so far explored only a limited spread of modalities and tasks, with generative AI appearing only once (14) and nurse-centered multimodal deployments remaining uncommon.

This gap between AI capability and ophthalmic nursing application represents a substantial research opportunity. The three triage studies in this review rely on classical machine learning and CNN (15–17), but recent research in general emergency departments has evaluated LLMs for triage tasks in large patient cohorts, without nursing engagement, which explains their exclusion from the current review (27). The ophthalmic nursing field is therefore well positioned to explore how these established approaches might be adapted and validated in nurse-led ophthalmic settings. Conversational AI for patient-facing health education has been extensively studied in other nursing domains, yet the ophthalmic nursing education literature has yielded only one such study (14) leaving substantial scope for purposefully designed trials. AR has been deployed in surgical and day ward contexts in this review (18, 20), and AR and virtual reality-based nursing education, widely reported in other specialties (28), presents a comparably unexplored but tractable direction in ophthalmic nursing.

### 4.4 The tertiary hospital gap: community and primary care settings

All seven included studies were conducted in tertiary academic hospitals or academic universities. No study examined AI applications in community health centers, primary care clinics, district hospitals, or homecare settings. This represents a gap in the published peer-reviewed English-language literature; whether the absence reflects a genuine lack of deployment or merely an absence of published evaluation studies is unclear. It is nonetheless a critical knowledge gap, given that community-based and primary care ophthalmic screening is precisely where the nursing workforce shortage is most acute, and where AI tools could most substantially expand access to care.

Established AI screening systems have demonstrated high accuracy in primary care and community settings (25, 29), but these programs have been evaluated primarily in terms of algorithm performance relative to specialist grading, without examining how nurses interact with these tools operationally. Future research should explicitly design nurse-led intervention studies in community settings, exploring how trained but non-specialist nurses can safely and effectively deploy AI-assisted ophthalmic screening, particularly for diabetic retinopathy and other conditions where early detection prevents irreversible vision loss.

### 4.5 Clinical governance, accountability, and professional identity

The deployment of AI in any clinical nursing context raises important governance questions that the included studies did not systematically address. None of the seven studies described protocols for clinical accountability when an AI system produces an erroneous output, including the criteria by which a nurse should override the AI recommendation, or the liability implications of AI-assisted versus nurse-only decision making. As AI tools move from proof-of-concept to routine deployment, these governance structures must be established prospectively and should form a required component of future evaluation studies. Relevant frameworks for AI governance in healthcare contexts are emerging (2), but their application to AI-assisted ophthalmic nursing practice requires discipline specific articulation.

One study in this review raises a particularly notable professional identity question. CRASCS (13), a pre-clinical robotic system designed to replicate the scrub nurse role in cataract surgery, is motivated explicitly by global surgical nursing shortages. The framing of robotic systems as substitutes rather than assistants for nursing roles deserves critical examination. Workforce displacement resulting from AI adoption has measurable consequences for nursing career development, professional identity, and the sustained supply of experienced clinical nurses. Future research on AI in ophthalmic surgical settings should explicitly examine these workforce implications alongside technical performance metrics, and where possible, involve practicing nurses as co-investigators in study design.

### 4.6 Strengths and limitations

To our knowledge, this is the first scoping review to draw on international, English-language sources to map AI applications in ophthalmic nursing. A related prior study was restricted to studies conducted in China and only searched Chinese databases through December 2023 (11). The present review is substantially complementary: two out of the three triage studies, the nursing education study, and the surgical robotics study included here were conducted in countries other than China (the United Kingdom, Turkey, and India), and four of the seven included studies were published after the prior review's search cutoff. Consequently, by synthesising multi-database literature, our study comprehensively characterizes the current evidence base, providing broader geographic coverage, more current evidence, and a systematic foundation for international research priority setting. By requiring all three themes (AI, ophthalmology, and nursing) to be simultaneously addressed, the review delineates a meaningful but undercharacterized research territory and provides a structured foundation for future study design and funding priorities.

Several limitations should be acknowledged. The evidence base is small (*n* = 7 studies), precluding quantitative synthesis and limiting the breadth of conclusions. The restriction to English-language publications may have omitted relevant research in Chinese or other languages. Gray literature (conference proceedings, preprints, governmental reports) was not searched, and relevant implementation studies outside the peer-reviewed literature may have been missed. All included studies were conducted in tertiary academic settings, limiting generalization. Two of three triage studies (15, 17) examined the same underlying DOTS system, and two workflow studies (18, 20) originated from a single institution, meaning the evidence landscape may be narrower in practice than the count of studies suggests. No included study reported nurse co-design or co-researcher involvement in AI development; this fifth potential engagement category, while consistent with participatory nursing research principles, was entirely absent from the evidence base. Finally, given the rapid pace of AI development, some of the systems described in the earlier included studies may have already been substantially updated or superseded.

## 5 Conclusions

This scoping review reveals a striking asymmetry: ophthalmology was among the first clinical domains to experience the transformative potential of AI, yet dedicated research on AI in ophthalmic nursing remains uncommon in the published literature. The seven included studies indicate meaningful proof-of-concept across triage, surgical assistance, day ward workflow, and nursing education, but the evidence base is small, heterogeneous, and concentrated in tertiary academic settings. Closing these gaps requires research that is explicitly nurse-centered, conducted in diverse care settings, and evaluated with outcomes that match nursing practice, including safety-sensitive triage performance, workflow impact, usability, and educational effectiveness.

## Data Availability

The original contributions presented in the study are included in the article/Supplementary Material, further inquiries can be directed to the corresponding author.
