# Supplementary material for: Current landscape and research gaps in artificial intelligence for ophthalmic nursing: a scoping review
Source: Front Med (Lausanne). 2026 Sep 3;13:1943007. doi: 10.3389/fmed.2026.1943007 (PMC13581657; doi:10.3389/fmed.2026.1943007)
Supplement: Supplementary file 1 [file Datasheet1.pdf]

Supplementary Appendix 1. Full Database Search Strategies

Search date: June 12, 2026. Four databases were searched from inception to June 12, 2026. Database-specific search strategies combining controlled vocabulary (MeSH where applicable) and keywords are provided below for each database.

Database: Web of Science Core Collections

(TI=("artificial* intelligence" OR "deep learning" OR "machine learning"
OR "neural network*" OR "natural language process*" OR "computer vision"
OR "edge intelligence" OR "ai agent*" OR "chatbot*" OR "robot*")
OR AK=("artificial* intelligence" OR "deep learning" OR "machine learning"
OR "neural network*" OR "natural language process*" OR "computer vision"
OR "edge intelligence" OR "ai agent*" OR "chatbot*" OR "robot*"))
AND
TS=("ophthal*" OR "ocular*" OR "eye disorder*" OR "eye disease*"
OR "eye injur*" OR "ametropia" OR "amblyopia" OR "anisocoria"
OR "cataract*" OR "anophthalmia" OR "dry eye syndrome"
OR "conjunctivitis" OR "conjunctiva*" OR "cornea*" OR "choroid*"
OR "extraocular muscle*" OR "eyelid*" OR "glaucoma*" OR "keratitis"
OR "kerato*" OR "macula*" OR "fundus image*" OR "diabetic retinopathy"
OR "optical coherence tomography" OR "retin*" OR "refractive error*"
OR "refractive disorder*" OR "strabismus" OR "trachoma*"
OR "vision defect*" OR "vision care" OR "vision disorder*"
OR "vision loss" OR "vision health" OR "vision screen*"
OR "visual care" OR "impaired vision" OR "visual defect*"
OR "visual health" OR "visual disorder*" OR "visual impair*"
OR "visual loss")
AND LA=(English)
AND DT=(Article OR Review)
AND (TI=("nurs*" OR "triage") OR AK=(nurs* OR "triage") OR WC=(Nursing))
NOT TS=("autonomous driving" OR "autonomous vehicle*" OR UAV OR drone*
OR "blind source separation" OR piglet* OR forest* OR tree* OR plant*
OR seed* OR rat OR rats OR mouse OR mice)

Database: PubMed

(
("artificial* intelligence"[ti] OR "machine learning"[ti]
OR "deep learning"[ti] OR "neural network*"[ti]
OR "computer vision"[ti] OR "natural language process*"[ti]
OR "edge intelligence"[ti] OR "chatbot*"[ti]
OR "ai agent*"[ti] OR "robot*"[ti])
OR
("artificial* intelligence"[ot] OR "machine learning"[ot]
OR "deep learning"[ot] OR "neural network*"[ot]
OR "computer vision"[ot] OR "natural language process*"[ot]
OR "edge intelligence"[ot] OR "chatbot*"[ot]
OR "ai agent*"[ot] OR "robot*"[ot])
)
AND

("ocular*"[tiab] OR "ophthal*"[tiab] OR "eye disease*”[tiab]
OR "eye disorder*"[tiab] OR "eye injur*"[tiab]
OR "amblyopia"[tiab] OR "ametropia"[tiab] OR "anisocoria"[tiab]
OR "anophthalmia"[tiab] OR "cataract*"[tiab]
OR "dry eye syndrome"[tiab] OR "cornea*"[tiab]
OR "conjunctiva*"[tiab] OR "conjunctivitis"[tiab]
OR "choroid*"[tiab] OR "eyelid*"[tiab]
OR "extraocular muscle*"[tiab] OR "glaucoma*"[tiab]
OR "kerato*"[tiab] OR "keratitis"[tiab] OR "macula*"[tiab]
OR "diabetic retinopathy"[tiab] OR "fundus image*"[tiab]
OR "optical coherence tomography"[tiab] OR "retin*"[tiab]
OR "refractive disorder*"[tiab] OR "refractive error*"[tiab]
OR "strabismus"[tiab] OR "trachoma*"[tiab]
OR "vision care"[tiab] OR "vision defect*"[tiab]
OR "vision disorder*"[tiab] OR "vision health"[tiab]
OR "vision loss"[tiab] OR "vision screen*"[tiab]
OR "impaired vision"[tiab] OR "visual care"[tiab]
OR "visual defect*"[tiab] OR "visual disorder*"[tiab]
OR "visual health"[tiab] OR "visual loss"[tiab]
OR "visual impair*"[tiab])
AND (english[la])
AND ("Journal Article"[pt] OR "Review"[pt])
AND ("nurs*"[ti] OR "triage"[ti] OR "nurs*"[ot] OR "triage"[ot]
OR "Nursing"[Mesh] OR "nursing"[Journal])
NOT
("autonomous driving"[tiab] OR "autonomous vehicle*"[tiab]
OR "UAV"[tiab] OR "drone*"[tiab]
OR "blind source separation"[tiab] OR "piglet*"[tiab]
OR "forest*"[tiab] OR "tree*"[tiab] OR "plant*"[tiab]
OR "seed*"[tiab] OR "rat"[tiab] OR "rats"[tiab]
OR "mouse"[tiab] OR “mice"[tiab])

Database: Scopus

(TITLE ("artificial* intelligence" OR "deep learning" OR "machine learning"
OR "neural network*" OR "natural language process*" OR "computer vision"
OR "edge intelligence" OR "ai agent*" OR "chatbot*" OR "robot*")
OR AUTHKEY ("artificial* intelligence" OR "deep learning" OR "machine learning"
OR "neural network*" OR "natural language process*" OR "computer vision"
OR "edge intelligence" OR "ai agent*" OR "chatbot*" OR "robot*"))
AND
TITLE-ABS-KEY ("ophthal*" OR "ocular*" OR "eye disorder*"
OR "eye disease*" OR "eye injur*" OR "ametropia" OR "amblyopia"
OR "anisocoria" OR "cataract*" OR "anophthalmia"
OR "dry eye syndrome" OR "conjunctivitis" OR "conjunctiva*"
OR "cornea*" OR "choroid*" OR "extraocular muscle*" OR "eyelid*"
OR "glaucoma*" OR "keratitis" OR "kerato*" OR "macula*"
OR "fundus image*" OR "diabetic retinopathy"
OR "optical coherence tomography" OR "retin*" OR "refractive error*"
OR "refractive disorder*" OR "strabismus" OR "trachoma*"
OR "vision defect*" OR "vision care" OR "vision disorder*"

OR "vision loss" OR "vision health" OR "vision screen*"
OR "visual care" OR "impaired vision" OR "visual defect*"
OR "visual health" OR "visual disorder*" OR "visual impair*"
OR "visual loss")
AND LANGUAGE (english)
AND (DOCTYPE (ar) OR DOCTYPE (re))
AND (TITLE ("nurs*" OR "triage") OR AUTHKEY ("nurs*" OR "triage")
OR SUBJAREA (nurs))
AND NOT
TITLE-ABS-KEY ("autonomous driving" OR "autonomous vehicle*"
OR "UAV" OR "drone*" OR "blind source separation" OR "piglet*"
OR "forest*" OR "tree*" OR "plant*" OR "seed*"
OR "rat" OR "rats" OR "mouse" OR “mice")

Database: CINAHL Complete

(TI ("artificial* intelligence" OR "deep learning" OR "machine learning"
OR "neural network*" OR "natural language process*" OR "computer vision"
OR "edge intelligence" OR "ai agent*" OR "chatbot*" OR "robot*")
OR AB ("artificial* intelligence" OR "deep learning" OR "machine learning"
OR "neural network*" OR "natural language process*" OR "computer vision"
OR "edge intelligence" OR "ai agent*" OR "chatbot*" OR "robot*")
OR SU ("artificial* intelligence" OR "deep learning" OR "machine learning"
OR "neural network*" OR "natural language process*" OR "computer vision"
OR "edge intelligence" OR "ai agent*" OR "chatbot*" OR "robot*"))
AND
("ophthal*" OR "ocular*" OR "eye disorder*" OR "eye disease*"
OR "eye injur*" OR "ametropia" OR "amblyopia" OR "anisocoria"
OR "cataract*" OR "anophthalmia" OR "dry eye syndrome"
OR "conjunctivitis" OR "conjunctiva*" OR "cornea*" OR "choroid*"
OR "extraocular muscle*" OR "eyelid*" OR "glaucoma*" OR "keratitis"
OR "kerato*" OR "macula*" OR "fundus image*" OR "diabetic retinopathy"
OR "optical coherence tomography" OR "retin*" OR "refractive error*"
OR "refractive disorder*" OR "strabismus" OR "trachoma*"
OR "vision defect*" OR "vision care" OR "vision disorder*"
OR "vision loss" OR "vision health" OR "vision screen*"
OR "visual care" OR "impaired vision" OR "visual defect*"
OR "visual health" OR "visual disorder*" OR "visual impair*"
OR "visual loss")
AND LA English
AND (PT Article OR PT Review)
AND (TI ("nurs*" OR "triage") OR AB ("nurs*" OR "triage")
OR SU ("nurs*" OR "triage"))
NOT
("autonomous driving" OR "autonomous vehicle*" OR "UAV" OR "drone*"
OR "blind source separation" OR "piglet*" OR "forest*" OR "tree*"
OR "plant*" OR "seed*" OR "rat" OR "rats" OR "mouse" OR "mice")
